# Enhanced Antioxidant Activity for Apple Juice Fermented with *Lactobacillus plantarum* ATCC14917

**DOI:** 10.3390/molecules24010051

**Published:** 2018-12-24

**Authors:** Zhongxi Li, Jing Teng, Yilu Lyu, Xiaoqian Hu, Yueliang Zhao, Mingfu Wang

**Affiliations:** 1 College of Food Science and Technology, Shanghai Ocean University, Shanghai 201306, China; ZX_L1992@163.com (Z.L.); victory2014t@163.com (J.T.); xqhu@shou.edu.cn (X.H.); 2 Laboratory of Quality and Safety Risk Assessment for Aquatic Products on Storage and Preservation (Shanghai), Ministry of Agriculture, Shanghai 201306, China; 3 Changle No. 2 Middle School, Changle, Pingdu 266714, Shandong Province, China; yilulyu163@163.com; 4 School of Biological Sciences, The University of Hong Kong, Pokfulam Road, Hong Kong 999077, China

**Keywords:** *Lactobacillus plantarum* ATCC14917 fermentation, antioxidant activity, apple phenolics, LC-MS/MS

## Abstract

The present study examined the effect of *Lactobacillus plantarum* ATCC14917 fermentation on the chemical composition and antioxidant activity of apple juice. Apple juice was fermented and examined of its antioxidant activity using chemical models and cellular antioxidant assay. Furthermore, the chemical composition of fermented apple juice was characterized by LC-MS/MS. *Lactobacillus plantarum* ATCC14917 fermentation showed an increase in DPPH and ABTS radical scavenging activity as well as cellular antioxidant activity of apple juice. However, fermentation decreased the total phenolic and flavonoid content. Subsequent LC-MS/MS analysis of the phenolic profile indicated that the content of 5-*O*-caffeoylquinic acid (5-CQA), quercetin, and phloretin with strong antioxidant activity was increased significantly after fermentation. The modified phenolic composition may contribute to the increased antioxidant activity of fermented apple juice. Our findings showed that *Lactobacillus plantarum* ATCC14917 fermentation may be an efficient way to enhance the bioavailability of phenolic compounds and to protect cells from oxidative stress.

## 1. Introduction

Apple, with pleasant taste and established nutritional value, is a fruit liked by people of different age groups [1]. The nutritional and organoleptic properties of apple are thought to be partly attributed by its rich polyphenol content [2]. Several classes of polyphenols have been found heterogeneously in apple tissues. Flavanol monomers (mainly (-)-epicatechin and (+)-catechin) and procyanidin (catechin oligomers and polymers) generally account for more than 80% of total apple polyphenols, followed by hydroxycinnamic acids (mainly caffeoylquinic acid), flavonols (mainly quercetin and quercetin glycosides), and dihydrochalcones (mainly phloridzin and phloretin xyloglucoside) [3,4]. Apple polyphenols have been reported to inhibit the proliferation of Caco-2 colon cancer cells [5], inhibit in vitro human low-density lipoprotein (LDL) cholesterol oxidation [6], protect against cigarette smoke-induced acute lung injury [7], and attenuate Alzheimer’s disease [8]. However, the low bioavailability of apple polyphenols can be an issue for utilization of them as disease preventive agents.

The absorption of polyphenols occurs mainly in the small intestine following the gastric and intestinal digestion phases in the gastrointestinal tract [9]. Soluble polyphenols are easily hydrolyzed and absorbed in the upper intestinal tract [10]. However, some of them still show low bioavailability. As an example, some apple polyphenols presenting in the forms of aglycones and glucoside conjugates (>90%) such as quercetin rhamnosides, quercetin xylosides, and quercetin galactosides, indeed have relative low bioavailability [11,12]. In addition, the insoluble bound polyphenols (polyphenols that are covalently bound with polysaccharides or proteins) cannot be absorbed at the small intestine as the soluble polyphenols do [13]. Thus, it is critical to increase the bioavailability of polyphenols to maximize their health benefits.

In recent decades, there has been an increasing interest in the bioconversion of phenolics by lactic acid bacteria (LAB). Some previous studies demonstrated that various LAB had the ability to de-carboxylate, de-esterify, de-methylate, and de-glycosylate dietary polyphenols [14,15,16,17,18,19]. As an example, LAB have been shown to be capable of causing biotransformation of polyphenols through the action of different glycosylhydrolases via the release of aglycones from glycol-conjugated phenolics [20,21]. Thus, with the aid of LAB, polyphenols can be biotransformed into compounds with enhanced bioavailability and bioactivity [22]. In addition, some polyphenols can be protected from chemical degradation under physiological conditions by LAB [23]. With the enhanced bioavailability of polyphenols, it is not a surprise to see the enhanced bioactivity for phenolic extracts pretreated with LAB. As an example, the antioxidant activities of some fruit and vegetable juices have been reported to be improved by fermentation using a few LAB strains, such as *L. plantarum* ASCC 292, *L. brevis* 145, *Bifidobacterium bifidum* CECT 870, *Weissella cibaria* 64, *Leuconostoc mesenteroides* 12b, *L. brevis* POM4, and *W. confusa* LK4 [24,25,26,27,28,29]. Thus, LAB fermentation could be chosen as an important way to improve the bioavailability of fruit polyphenols.

This study aimed to investigate the capacity of a specific strain of LAB, *L. plantarum* ATCC14917, to enhance the antioxidant activity of apple juice. Furthermore, with the aid of mass spectrometric technique, the phenolic profiles of fermented apple juice were studied.

## 2. Results and Discussion

### 2.1. Changes in Lactobacillus plantarum Counts, pH, and Glucose and Fructose Content during Apple Juice Fermentation with L. plantarum ATCC14917

The cell growth profile of *L. plantarum* ATCC14917 during apple juice fermentation was shown in Table 1. Due to the adequate nutrient content and suitable growth conditions, a rapid growth of *L. plantarum* was observed during the first 24 h of fermentation. After 24 h, population growth slowed down. *L. plantarum* reached a maximum viable cell number of 8.37 log ± 0.34 log CFU/mL at 48 h of fermentation. A drop in the total microbial population was observed after 48 h of fermentation, which may be due to the fact that *L. plantarum* metabolized carbohydrates to lactic acid and thus caused the decreased pH value of the growth environment [30]. As expected, the pH of apple juice dropped from 6.2 to 3.68 after 72 h fermentation (Table 1). Kandler et al. [31] reported that glucose and fructose were suitable energy and carbon sources for Lactobacillus strains. The changes of carbohydrates of apple juice were measured during fermentation with *L. plantarum*. As shown in Table 1, the major detected sugars in apple juice were glucose and fructose. Glucose content decreased from 11.66 to 6.22 mg/mL after 72 h fermentation. Fructose content decreased from 23.89 to 16.09 mg/mL after 72 h fermentation. The decreased pH value could be attributed to sugar consumption and subsequent acid production. Consistent with our findings, Kaprasob et al. [32] reported that *L. acidophilus*, *L. casei*, and *L. plantarum* caused decreased pH of cashew apple juice, which resulted in the inhibited proliferation of *L. acidophilus*, *L. casei*, *and L. plantarum* cells during fermentation. Espirito-Santo [33] found that the pH of apple juices decreased significantly from 3.7 to 3.4 after 50 h fermentation with *L. acidophillus* L10, *L. casei* L26, *L. paracasei* L33, *L. plantarum* 299v, and *L. rhamnosus* LGG ATCC 53103. The counts of these LAB strains were inversely and strongly correlated to pH in apple juices during the fermentation process. 

### 2.2. Changes in Total Antioxidant Capacity of Apple Juice Before and after L. plantarum ATCC14917 Fermentation

Table 1 shows the antioxidant capacity of apple juice determined by DPPH assay and ABTS assay. *L. plantarum* fermentation for 24 h significantly enhanced the DPPH and ABTS scavenging activity of apple juice by 23% and 28%, respectively. From 24 h to 72 h, the DPPH scavenging activity of fermented apple juice slightly decreased from 48.18% to 43.95% (*p* < 0.05)) and the ABTS scavenging activity of fermented apple juice remained stable (*p* > 0.05). The overall antioxidant activity should be the result of joint contribution of multiple antioxidants in apple juice [34]. The increased antioxidant activity may be caused by *L. plantarum* fermentation-induced changes of total phenolics content (TPC) and total flavonoid content (TFC). *L. plantarum* WCFS1 and *L. plantarum* ATCC SD5209 fermentation have been reported to enhance the antioxidant capacity of fruit and vegetable juice with increased TPC and TFC [35,36].

### 2.3. Changes in Total Phenolic Content (TPC) and Total Flavonoid Content (TFC) During Apple Juice Fermentation

Whether *L. plantarum* fermentation increased the TPC and TFC content was then tested. TPC determined by Folin–Ciocalteu method and TFC determined using the aluminum chloride colorimetric method were presented in Table 1. Surprisingly, there was a dramatic decrease in both TPC and TFC of the apple juice during the fermentation process. TPC in fermented apple juice dropped from 115.6 to 89.3 μg/mL. TFC in fermented apple juice dropped from 119 to 77.7 μg/mL, which is equivalent to a reduction yield of 22% and 34.8%, respectively. Another study also reported that TPC and TFC of olives decreased during fermentation with *L. plantarum* PTCC 1058 [37,38]. The reduced TPC and TFC can be explained by the fact that the presence of lactic acid bacteria contributed to simple phenolic conversion and the depolymerisation of high molecular weight phenolic compounds in apple juice [39]. In addition, the reduced TPC and TFC did not contradict with the enhanced antioxidant activity of fermented apple juice, because lactic acid bacteria might deplete the available glucose molecule in the phenolic compounds, resulting in the production of free aglycones with higher number of hydroxyl groups or lower steric hindrance to hydroxyl groups [40], which led to some metabolites with higher antioxidant activity being produced during fermentation of the apple juice [37,39]. To confirm the hypothesis, the phenolic profile of apple juice before and after *L. plantarum* fermentation was then determined.

### 2.4. Changes in Apple Phenolic Profile Before and After L. plantarum Fermentation

The specific phenolic compounds and their contents in the fermented and nonfermented apple juice were measured by LC–MS/MS analysis. Phenolic compounds in apple juice were identified by comparing retention time and mass spectra with that of commercial standards. In cases of unavailable standards, compounds were putatively identified based on their exact mass of [M − H]^−^ ions, tandem mass spectrometric fragmentation patterns, and MS data published earlier [24,41]. The phenolic profile of apple juice fermented for 24 h with nonfermented apple juice was compared. As shown in Table 2, 13 phytochemicals including quinic acid, gallic acid, 3-*O*-caffeoylquinic acid (3-CQA), 5-*O*-caffeoylquinic acid (5-CQA), caffeic acid, (+)-catechin, epicatechin, ellagic acid, quercetin-3-*O*-galactoside, quercetin-3-*O*-glucoside, quercetin-3-*O*-xyloside, phlorizin, and quercetin were detected in nonfermented apple juice, with quinic acid and quinic acid ester derivatives, quercetin and its glycosides quercetin-3-*O*-galactoside and quercetin-3-*O*-xyloside as the major ones. After 24 h fermentation, quinic acid, quercetin-3-*O*-galactoside, quercetin-3-*O*-glucoside, and phlorizin were greatly metabolized by *L. plantarum* ATCC14917, resulting in the increased content of 5-CQA, quercetin, and phloretin which were with strong antioxidant activity. Consistent with our results, *L. plantarum* ATCC14917 has been reported to remove sugar moieties and hydrolyzed galloyl moieties of a variety of phenolic compounds during fermentation, resulting in changes in phenolic profiles [42,43]. The enhanced antioxidant activity of apple juice by *L. plantarum* fermentation could be attributed to the changed phenolic profile.

### 2.5. Influence of L. plantarum Fermentation on Cellular Antioxidant Activity of Apple Juice Extract in RAW264.7 Cells

**Figure 1:**
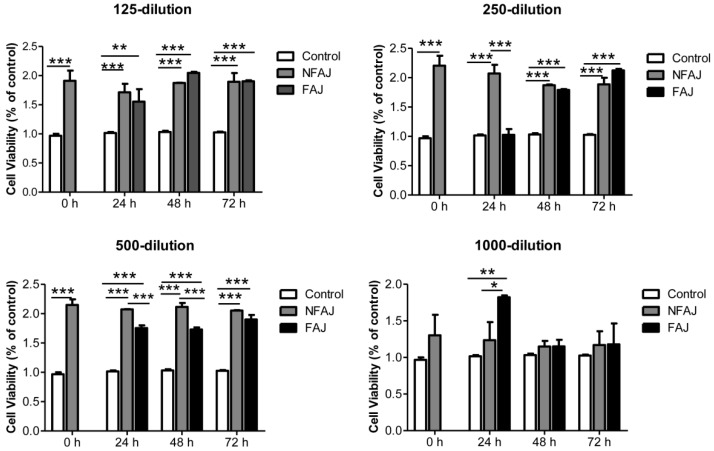
Cell viability of RAW 264.7 treated with nonfermented apple juice (NFAJ) and fermented apple juice (FAJ). The apple juice extract was diluted for 125, 250, 500, and 1000 times. Cells were treated with or without the diluted apple juice extract for 24 h and cell viability was assessed by MTT assay. *n* = 3, * *p* < 0.05, ** *p* < 0.01, and *** *p* < 0.001.

Raw264.7 cells-based model was used to evaluate the cellular antioxidant activity (CAA) of apple juice. The CAA assay takes into account the bioavailability, distribution, and metabolism of antioxidants in live cells, which was a better method for assessment of antioxidant activity than chemical methods [44]. In the present study, the effect of nonfermented apple juice and fermented apple juice extract on Raw264.7 cells viability was firstly measured using MTT assay. As shown in Figure 1, the apple juice extract diluted for 125, 250, 500, and 1000 times promoted Raw264.7 cells growth well. Apple juice extract diluted for 125 times were used for subsequent CAA assay. As shown in Table 3, after 24 h fermentation, the CAA value of apple juice was about doubled, indicating that fermentation with *L. plantarum* could improve the uptake and bioavailability of antioxidant ingredients of apple juice. The enhancement of CAA may be due to the fact that bacterial metabolism, mainly deglycosylation and degallation activities, released free aglycones with higher number of hydroxyl groups or lower steric hindrance to hydroxyl groups [24]. In addition, as expected, the CAA results agreed well with antioxidant capacity determined by non-cell model as described in Section 2.2.

It can be concluded from these results that *L. plantarum* fermentation improved the antioxidant activity of apple juice considerably. According to the results described in previous Sections, the majority of the quercetin and phloretin were conjugated through hydroxyl groups with sugars and glycosides (present in the form of quercetin-3-*O*-galactoside, quercetin-3-*O*-glucoside, and phlorizin) in apple juice. The glucose molecule available in the quercetin and phloretin structure was likely depleted by *L. plantarum* as an energy source, resulting in the production of related aglycones with higher radical scavenging effect, which led to some metabolites such as quercetin and phloretin with strong antioxidant activity being produced during the fermentation. The increased content of gallic acid, 3-CQA, and 5-CQA after *L. plantarum* fermentation also contributed to the enhanced cellular antioxidant activity of apple juice. Consistent with our results, LAB fermentation increased the antioxidant activities of cashew apple juice during the beginning of fermentation (12 h), which was thought to be associated with the production of vitamin C and phenolic compounds having antioxidant capacity. It is believed that oxidative stress is involved in the pathogenesis of a number of chronic diseases [45]. Our study showed that lactic acid bacteria fermentation could enhance the bioavailability and antioxidant capacity of apple juice. Lactic acid bacteria-fermented apple juice could be served as a kind of functional food to relieve free radical species-induced disease.

## 3. Materials and Methods

### 3.1. Microorganisms and Culture Conditions

*Lactobacillus plantarum* ATCC14917 obtained from Taiwan Yaxin Biotechnology Co., Ltd. was maintained at −80 °C in MRS broth supplemented with glycerol (60% *v/v*). For activation, 10 mL of sterile MRS were inoculated with 10% (*v/v*) of organism and incubated at 37 °C for 24 h. After the second transfer in MRS broth, the activated microorganisms were used for apple juice fermentation.

### 3.2. Preparation of Apple Juice

Apples (Hua Niu) were purchased from a local fruit market (Shanghai, China). The fruits were washed with tap water, dried with paper towels, cut into strips to remove seeds, stems, and over-ripened portions. A quantity of 700 g apple and 1000 mL water were mixed and apple juice was obtained using a juicer (Joyoung JYL-Y5, Joyoung Co., Ltd., Jinan, Shandong, China). The apple juice was then filtered using a cotton cloth filter and stored at −20 °C (maximum 2 weeks) prior to use. Three independent experiments were performed.

### 3.3. Fermentation of Apple Juice Using Lactobacillus plantarum ATCC14917

The initial pH of apple juice from Section 2.2 was adjusted to 6.2 with 2 M NaOH [46] and pasteurized at 80 °C for 5 min. A 2% (*v/v*) of *L. plantarum* was allotted into 250 mL Erlenmeyer flasks containing 150 mL of the pasteurized juice, and incubated at 37 °C for 24, 48, and 72 h. Viable cells, glucose and fructose content, and pH values were determined at 24, 48, and 72 h of fermentation. Nonfermented apple juice was incubated under the same conditions and used as the control.

### 3.4. Determination of Viable Cells

Viable cells of *L. plantarum* ATCC14917 were determined by the standard plate count method. Serial dilutions (with peptone water) of fermented apple juice were prepared. Aliquots of 0.1 mL of appropriate dilutions were plated in MRS agar (pH 6.2 ± 0.2) plates by the spread plate method. The plates were incubated at 37 °C for 48 h. Plates containing 20–350 colonies were measured and recorded as colony forming units (CFU) per mL of solution. 

### 3.5. Determination of Glucose and Fructose Content and pH Values

Fructose and glucose of fermented or nonfermented apple juice were determined according to the method described previously [47] using HPLC (Waters 2695 series, Waters Corporation, Milford, MA, USA) equipped with a G1315B refractive index detector (RID). A Cosmosil Sugar-d column (250 mm × 4.6 mm) was employed for separation. Acetonitrile-water (75%:25%) was used as the mobile phase. The flow rate of the mobile phase was 1.0 mL/min. Then, 20 μL of fermented or nonfermented apple juice was injected to the HPLC. Fructose and glucose contents were reported using external standards.

The fermented or nonfermented apple juice pH was determined by direct measure in a Five Easy FE20 portable pH meter (METTLER TOLEDO International Trading Co. Shanghai, China).

### 3.6. Preparation of Crude Methanol Extract of Fermented Apple Juice

An amount of 30 mL of fermented apple juice or nonfermented apple juice was mixed with 60 mL of methanol, sonication for 30 min (40 °C), and centrifuged at 8000× *g* for 10 min to get the supernatants. The supernatants were filtered through 0.45 μM hydrophilic filter (ANPEL Scientific Instrument Co., Shanghai, China) and used as extract for determining DPPH radical scavenging activity, ABTS radical scavenging activity, total phenolic content (TPC), total flavonoid content (TFC), and cellular antioxidant activity.

### 3.7. Antioxidant Activity of Fermented Apple Juice

#### 3.7.1. DPPH Radical Scavenging Activity

The ability of fermented apple juice to scavenge 1, 1-diphenyl-2-picryl-hydrazyl (DPPH) radical was determined according to a published method [48] with slight modifications. In brief, 1 mL of fermented or nonfermented apple juice extract from Section 3.6 was mixed with 2 mL of methanolic solution of DPPH (0.045 mg/mL). Absorbance was measured at 517 nm using a UV-754N ultraviolet spectrophotometer (INESA Analytical Instrument Co., Ltd. Shanghai, China) after the mixture was kept in the dark for 30 min. Radical scavenging ability (RSA) was expressed in terms of percent DPPH inhibition using the following formula:

DPPH RSA (%) = [(A_0_−A_s_)/A_0_] × 100 (1)

where A_0_ is the absorbance of DPPH radical solution without sample and A_s_ is the absorbance of the sample.

#### 3.7.2. ABTS Radical Scavenging Activity

ABTS radical scavenging activity of fermented apple juice was measured by the ABTS cation decolorization assay [49] with slight modifications. The ABTS radical cation (ABTS•+) was produced by reaction of 7 mM ABTS solution with 2.45 mM potassium persulphate. The mixture was put in the dark for 12 h at room temperature before use. The ABTS•+ solution was diluted with ethanol to achieve an absorbance of 0.70 ± 0.02 at 734 nm. Next, 300 μL of fermented/nonfermented apple juice extract from Section 3.6 was allowed to react with 5 mL of the diluted ABTS•+ solution and absorbance was measured on a UV-754N ultraviolet spectrophotometer (INESA Analytical Instrument Co., Ltd. Shanghai, China) at 734 nm after 6 min. RSA was expressed in terms of percent ABTS inhibition using the following equation:

ABTS RSA (%) = [(A_0_−A_s_)/A_0_] × 100 (2)

where A_0_ is the absorbance of ABTS radical solution without sample and A_s_ is the absorbance of the sample.

### 3.8. Phytochemical Content Analysis

#### 3.8.1. Total Phenolic Content

The total phenolic content (TPC) of apple juice was determined using the Folin–Ciocalteu method [50]. Briefly, 1 mL of the fermented/nonfermented apple juice extract from Section 3.6 was added to 5 mL of Folin–Ciocalteu reagent. After standing at room temperature for 3 min, 4 mL of sodium carbonate (7.5% *w*/*v*) was added to the mixture. Then the samples were allowed to stand at room temperature for 60 min, the absorbance of the mixture was measured at 765 nm on a UV-754N ultraviolet spectrophotometer (INESA Analytical Instrument Co., Ltd. Shanghai, China). TPC was expressed as gallic acid equivalents (GAE) (μg GAE/mL).

#### 3.8.2. Total Flavonoid Content

The total flavonoid content (TFC) of apple juice was determined using the aluminum chloride (AlCl_3_) colorimetric method [51]. Briefly, 300 μL NaNO_2_ (50 g/L) and 4 mL distilled water were added to 1 mL fermented/nonfermented apple juice extract from Section 3.6. The mixture was allowed to stand for 5 min at room temperature. After that, 1 mL of AlCl_3_ (100 g/L) was added to the mixture, vortexed, and allowed to stand for 5 min. Then, 2 mL NaOH (1 mol/L) and 2.4 mL of distilled water were added to the mixture and allowed to stand for 10 min. Absorbance of the mixture was determined at 510 nm using a UV-754N ultraviolet spectrophotometer (INESA Analytical Instrument Co., Ltd. Shanghai, China). The TFC was expressed as rutin equivalent (RE) (μg RE/mL).

### 3.9. Chromatographic Profiling of Polyphenols in Apple Juice

A 10 mL quantity of fermented/nonfermented apple juice extract from Section 3.6 was freeze-dried and 0.05 g of lyophilized powder was dissolved in 1 mL of methanol, sonication for 30 min (40 °C), filtered through 0.22 μm hydrophilic filter, and collected for analysis. The analysis of polyphenols in apple juice was performed on a Waters 2695 HPLC system with a DAD detector and interfaced to an AB SCIEX 4500 QTRAP LC–MS/MS System. The separation of compounds was carried out on a C_18_ column (250 mm × 4.6 mm, 5 μm, Phenomenex, Torrance, CA, USA). The mobile phase composed of 0.1% (*v/v*) formic acid in water (solvent A) and acetonitrile (solvent B) of the following gradients: 0 min, 5% B:95% A; 5 min, 5% B: 95% A; 30 min, 45% B: 55% A; 45 min, 80% B: 20% A; 55 min, 80% B: 20% A; 55.1 min, 5% B: 95% A; 60 min, 5% B: 95% A. Injections were carried out with an autosampler (4 °C) and the injection volume was 10 μL. The flow rate was 1 mL/min for a total run time of 60 min [52]. The MS parameters were as follows: negative ion mode; spray voltage, −4500 V; scan range, *m/z* 100 to 800 Da; ion source temperature, 550 °C; declustering potential (DP): −68 V; collision energy (CE): −15 eV; entrance potential (EP): −10 eV. Identification of the phenolics was carried out by comparing retention time and mass spectra with that of commercial standards. Available standards included Gallic acid (G7384), (+)-catechin (43412), caffeic acid (C0625), chlorogenic acid (C3878), ellagic acid (E2250), and quercetin-3-*O*-galactoside (00181585) were obtained from ANPEL Scientific Instrument Co. Shanghai, China. Quercetin (Q4951), phlorizin (79589), and phloretin (P7912) were purchased from Sigma Chemical Co., St. Louis, MO, USA. In cases of unavailable standards, compounds were putatively identified based on their exact mass of [M − H]^−^ ions, tandem mass spectrometric fragmentation patterns, and MS data published earlier [53,54].

### 3.10. Cell Culture and Cell Viability Test

Mouse Raw264.7 macrophages, purchased from American Type Culture Collection (ATCC, Rockville, MD, USA), were cultured in DMEM supplemented with 10% FBS plus 1% Penicillin-Streptomycin at 37 °C under 5% carbon dioxide. Cells used in this study were between 4 and 20 passages. Cell viability was measured by the crystal violet assay [55]. Briefly, cells were seeded in 96-well microtiter plates at a density of 1 × 10^4^ cells per well for 24 h. The fermented/nonfermented apple juice extracts from Section 3.6 were diluted with fresh complete medium for 125, 250, 500, and 1000 times to figure out a noncytotoxic concentration for the cellular antioxidant activity test. Cells were treated with or without the apple juice extract for 24 h. The medium was then discarded and cells were washed with PBS before adding 50 μL of 0.5% crystal violet for 6 min. After washing twice with tap water, the plates were dried in ambient temperature. Then 200 μL of methanol were added in each well. The optical density was measured at 570 nm by a PMT49984 Multilabel Plate Reader (BioTek Instruments, Inc., Winooski, VT, USA) and the results were calculated as the percentage of blank.

### 3.11. Cellular Antioxidant Activity (CAA) Assay

The CAA assay was performed according to a published method [44] with slight modification. Briefly, Raw264.7 cells were seeded in 96-well microplates at a density of 1 × 10^4^/well for 24 h. The cells were then treated with fermented/nonfermented apple juice extract (from Section 3.6) that were diluted with fresh complete medium for 125 times for 2 h. Cells were then washed with PBS and stained with 25 μM DCFH-DA at 37 °C for 1 h. Then, the cells were rinsed with PBS for three times. Next, 100 μL of ABAP (300 μM) was added to the cells and the fluorescence was recorded using a PMT49984 Multilabel Plate Reader (BioTek Instruments, Inc. USA) at 37 °C. Emission at 535 nm was measured with excitation at 485 nm every 3 min for 1 h. Control wells were treated with DCFH-DA and ABAP, without 2 h pre-incubation of fermented/nonfermented apple juice extract. Blank wells were treated with DCFH-DA only. The sample background wells were treated with fermented/nonfermented apple juice extract and DCFH-DA with ABAP.

After blank subtraction from the fluorescence readings, the area under the curve of fluorescence versus time was integrated by Origin 9.1 (OriginLab, Northampton, MA, USA) to calculate the CAA value as follows:

CAA unit = 100 × (1−A_S_/A_C_) (3)

where A_S_ is the integrated area under the sample fluorescence versus time curve and A_C_ is the integrated area from the control curve.

### 3.12. Statistical Analysis

Results were expressed as mean ± standard deviation of at least three independent experiments. Statistical analysis was carried out using ANOVA by Prism 5.0, GraphPad Software (GraphPad Software Inc., La Jolla, CA, USA). The significance of differences was calculated using the Student’s paired t-test. *p*-value < 0.05 was considered statistically significant.

## 4. Conclusions

In summary, *L. plantarum* ATCC14917 fermentation modified the phenolic composition of apple juice and enhanced its overall antioxidant capacity. Lactic acid bacteria fermentation may be employed as a simple and valuable biotechnology to enhance the bioavailability of polyphenols of apples. The fermented apple juice could be consumed as a kind of functional food to relieve free radical species-induced disease.

## Figures and Tables

**Table 1:** Effect of fermentation on *Lactobacillus plantarum* counts, pH value, glucose content, fructose content, DPPH radical scavenging activity, ABTS radical scavenging activity, total phenolics content (TPC), and total flavonoid content (TFC) of apple juice. NFAJ: Nonfermented juice; FAJ: fermented apple juice.

Sample	pH	*Lactobacillus* Counts (log CFU/mL)	Fructose (mg/mL)	Glucose (mg/mL)	DPPH RSA (%)	ABTS RSA (%)	TPC (μg GAE/mL)	TFC (μg RE/mL)
0 h-NFAJ	6.2 ± 0 ^d^	6.48 ± 0.04 ^a^	23.89 ± 0.2 0 ^b^	11.66 ± 0.76 ^c^	24.95 ± 0.86 ^a^	41.99 ± 16.41 ^a^	115.61 ± 1.82 ^b^	119.21 ± 2.07 ^d^
24 h-NFAJ	6.2 ± 0.03 ^d^		23.31 ± 0.78 ^b^	11.06 ± 1.18 ^bc^	25.45 ± 0.16 ^a^	39.07 ± 4.22 ^a^	115.62 ± 1.91 ^b^	119.21 ± 1.59 ^d^
24 h-FAJ	4.36 ± 0.01 ^c^	7.8 ± 0.14 ^b^	20.41 ± 1.52 ^ab^	10.85 ± 1.13 ^bc^	48.18 ± 1.94 ^d^	70.4 ± 8.01 ^c^	111.29 ± 3.21 ^b^	104.03 ± 5.53 ^c^
48 h-NFAJ	6.2 ± 0.01 ^d^		23.27 ± 0.58 ^b^	11.46 ± 1.04 ^bc^	25.95 ± 0.86 ^a^	42.65 ± 0.93 ^a^	115.58 ± 1.85 ^b^	115.87 ± 7.84 ^d^
48 h-FAJ	3.93 ± 0 ^b^	8.37 ± 0.34 ^c^	17.63 ± 2.21 ^a^	9.43 ± 0.13 ^b^	37.89 ± 0.63 ^b^	57.02 ± 7.79 ^b^	90.21 ± 1.77 ^a^	92.93 ± 4.42 ^b^
72 h-NFAJ	6.2 ± 0.01 ^d^		22.39 ± 0.91 ^b^	11.36 ± 0.47 ^bc^	25.64 ± 0.11 ^a^	41.19 ± 0.56 ^a^	115.28 ± 1.43 ^b^	118.54 ± 1.8 ^d^
72 h-FAJ	3.68 ± 0.01 ^a^	7.85 ± 0.22 ^b^	16.09 ± 3.76 ^a^	6.22 ± 0.43 ^a^	43.95 ± 0.95 ^c^	70.0 ± 0.47 ^c^	89.33 ± 4.39 ^a^	77.7 ± 2.74 ^a^

Values are mean ± standard deviation (*n* = 3). Values within treatments in a column with different superscript lowercase letters (a–d) differ significantly (*p* < 0.05).

**Table 2:** Retention time (Rt) and mass spectral data for tentative identification of polyphenols in apple juice (NFAJ: Nonfermented apple juice; FAJ: fermented apple juice).

Rt (min)	[M − H]^−^ (*m/z*)	MS/MS (*m/z*)	Compound	NFAJ (μg/mL)	FAJ (μg/mL)	Changes (%)
3.38 and 4.01	191	173, 127	Quinic acid	N.A.	N.D.	Decrease
14.38	179	135	Caffeic acid	1.02	0.67	Decrease 34.3%
14.98	289	245	(+)-Catechin	7.3	5.3	Decrease 27.4%
15.0	289	245, 205	Epicatechin	N.A.	N.A.	Decrease 33.3%
17.35	301	185	Ellagic acid	0.78	0.39	Decrease 49.7%
18.01	463	300, 301	Quercetin-3-*O*-galactoside	4.19	2.39	Decrease 43.0%
18.32	463	300, 301	Quercetin-3-*O*-glucoside	N.A.	N.D.	Decrease
21.76	435	273, 125	Phlorizin	2.69	0.97	Decrease 63.9%
4.61	169	125	Gallic acid	0.70	1.42	Increase 102.9%
13.18	353	191, 179, 135	3-*O*-caffeoylquinic acid	N.A.	N.A.	Increase 121.4%
13.68	353	191, 179	5-*O*-caffeoylquinic acid	2.98	21.2	Increase 611.4%
18.64	353	173, 179	Quinic acid conjugate	N.D.	N.A.	Increase
24.54	301	179, 151	Quercetin	0.76	0.93	Increase 22.4%
28.61	273	167	Phloretin	N.D.	0.84	Increase
19.73	433.1	301	Quercetin-3-*O*-xyloside	N.A.	N.A.	No change

N.A., Standards were unavailable. The compounds’ changes (%) after fermentation were calculated based on the changes of peak area. N.D., not detectable.

**Table 3:** Cellular antioxidant activity of fermented/nonfermented apple juice extract. NFAJ: Nonfermented juice; FAJ: fermented apple juice.

Sample	CAA Unit (%)
0 h-NFAJ	21.63 ± 5.28 ^a^
24 h-NFAJ	22.29 ± 6.39 ^a^
24 h-FAJ	52.28 ± 9.90 ^b^
48 h-NFAJ	20.30 ± 5.63 ^a^
48 h-FAJ	54.35 ± 8.87 ^b^
72 h-NFAJ	20.96 ± 5.87 ^a^
72 h-FAJ	58.55 ± 7.19 ^b^

Values are mean ± standard deviation (*n* = 3). Values within treatments in a column with different superscript lowercase letters (a, b) differ significantly (*p* < 0.05).

## Abbreviations

LAB: lactic acid bacteria; TPC: total phenolics content; TFC: total flavonoid content; RSA: radical scavenging activity; 3-CQA: 3-*O*-caffeoylquinic acid; 5-CQA: 5-*O*-caffeoylquinic acid; Rt: retention time; NFAJ: nonfermented juice; FAJ: fermented apple juice; CAA: cellular antioxidant activity; CFU: colony forming units; RID: refractive index detector; GAE: gallic acid equivalents; RE: rutin equivalent; DPPH: 1, 1-diphenyl-2-picryl-hydrazyl; ABTS: 2, 2′-azino-*bis*(3-ethylbenzothiazoline-6-sulfonic acid); DMEM: Dulbecco’s modified Eagle medium; FBS: fetal bovine serum; DCFH-DA: 2′, 7iadichlorodihydrofluorescein diacetate; ABAP: 2,2-Azobis(2-amidinopropane); MTT:3-(4,5-dimethyl-2-thiazolyl)-2,5-diphenyl-2-H-tetrazolium bromide.
